# Model checking optimal finite-horizon control for probabilistic gene regulatory networks

**DOI:** 10.1186/s12918-017-0481-6

**Published:** 2017-12-14

**Authors:** Ou Wei, Zonghao Guo, Yun Niu, Wenyuan Liao

**Affiliations:** 1 Department of Computer Science, Nanjing University of Aeronautics and Astronautics, Nanjing, China; 2 Department of Mathematics and Statistics, University of Calgary, Calgary, Canada

**Keywords:** Probabilistic Boolean networks, Context-sensitive, Perturbation, Optimal control, Model checking

## Abstract

**Background:**

Probabilistic Boolean networks (PBNs) have been proposed for analyzing external control in gene regulatory networks with incorporation of uncertainty. A context-sensitive PBN with perturbation (CS-PBNp), extending a PBN with context-sensitivity to reflect the inherent biological stability and random perturbations to express the impact of external stimuli, is considered to be more suitable for modeling small biological systems intervened by conditions from the outside. In this paper, we apply probabilistic model checking, a formal verification technique, to optimal control for a CS-PBNp that minimizes the expected cost over a finite control horizon.

**Results:**

We first describe a procedure of modeling a CS-PBNp using the language provided by a widely used probabilistic model checker PRISM. We then analyze the reward-based temporal properties and the computation in probabilistic model checking; based on the analysis, we provide a method to formulate the optimal control problem as minimum reachability reward properties. Furthermore, we incorporate control and state cost information into the PRISM code of a CS-PBNp such that automated model checking a minimum reachability reward property on the code gives the solution to the optimal control problem. We conduct experiments on two examples, an apoptosis network and a WNT5A network. Preliminary experiment results show the feasibility and effectiveness of our approach.

**Conclusions:**

The approach based on probabilistic model checking for optimal control avoids explicit computation of large-size state transition relations associated with PBNs. It enables a natural depiction of the dynamics of gene regulatory networks, and provides a canonical form to formulate optimal control problems using temporal properties that can be automated solved by leveraging the analysis power of underlying model checking engines. This work will be helpful for further utilization of the advances in formal verification techniques in system biology.

## Background

Research on biological regulation is important for understanding cellular functions of organisms and developing therapeutic interventions in medical applications. One of the central issues in this area is finding optimal control for gene regulatory networks [1]. At a given state of the network, by imposing external interventions, e.g., drugs, radiation, and chemo-therapy, the expression status of particular genes in the network can be changed; therefore, the whole network can transition to a desirable state or a steady-state distribution via the interaction between genes. The external inputs provided during the process thus form a control policy for regulating cell behaviors of organisms. Optimal control problem for gene regulation is typically concerned with finding control policies that minimize a cost function in a treatment horizon.

Probabilistic Boolean networks (PBNs) [2], an extension of Boolean networks (BNs),enable effectively expression of rule-based dependencies between genes and representation of the switching behaviors of genetic process, and have been been widely used by system biologists in external control for gene regulatory networks with uncertainty [3]. A BN is a discrete, deterministic model, where the expression status of each gene is represented by a binary value: 0 (off) or 1 (on). Each node in a BN is associated with a Boolean function that, defined over the expression status of the corresponding gene and other ones, describes the interactions between them. To model uncertainty in realistic biological systems, PBNs have been developed as an extension of BNs. In a PBN, several Boolean functions are defined for each gene, and the functions are chosen randomly with respect to a given probability distribution at each time step. Unlike a BN, such a PBN, called an *instantaneously random* PBN, therefore is a non-deterministic model, which essentially represents a set of BNs such that a governing one is randomly decided at each time to instantiate the PBN.

In this paper, we investigate the optimal control problem for a *context-sensitive* PBN with *perturbation* (CS-PBNp). The goal of introducing context-sensitivity to a PBN is to characterize the inherent stability of biological systems [4], which is achieved by imposing a probability *q* when the switching in the Boolean networks is decided; Thus, different from that in an instantaneously random PBN, the switching behavior between the governing Boolean networks in a context-sensitive PBN is restricted. A PBN subject to random perturbation, on the other side, enables capturing the impact of external stimuli to the genome from the outside, such as mutagens and heat stress [5]. Due to the effect of random perturbations, the value of each gene in a PBN can be flipped with a small perturbation probability *p*; as a result, the PBN corresponds to an ergodic Markov chain, where all the states communicate and there exists a unique steady-state distribution. As indicated in [4], a CS-PBNp is believed to be more appropriate for modeling small biological systems where the behavior of the genes in the network is moderated by external conditions.

We provide an approach based on the technology of probabilistic model checking for solving optimal control problem for a CS-PBNp. Model checking [6] is a formal verification technique for automated verifying behavioral properties of concurrent systems based on state-exploration. In this method, a modeling language is provided for describing the behavior of a system composed of synchronous or asynchronous components. The desired properties of the system are formulated using temporal logic formulas equipped with formal syntax and semantics. Thanks to the efficiency of the underlying data structures and algorithms, a model checker can automatically build state transition relations of the system, and conducts an exhaustive search of the system’s statespace to check whether the behavior specified by a temporal property holds on the system or not. Recently, to support modeling and verification of systems with random and probabilistic behavior, probabilistic model checking has been developed, which extends traditional model checking with advanced numerical computation algorithms, temporal logic formulas and structures for describing probability and cost/reward information, providing techniques for *automatically* analyzing a wide class of quantitative features about system behavior.

Applying (probabilistic) model checking to analysis of biological behavior has been greatly studied in system biology [7–10]. Specifically, in [11, 12], Kobayashiy and Hiraishi proposed to use probabilistic model checking to solve optimal control problems for (context-sensitive) PBNs. Unlike other optimal control approaches for PBNs, e.g., [1, 4, 13–15], that are usually developed over integer programming and dynamic programming, the method based on probabilistic model checking does not need explicit computation of large-size state transition relations associated with PBNs and offers a framework for flexible specification and analysis. The methods presented in [11, 12], however, have weakness: they can not model impact of random perturbations on PBNs; moreover, they did not take take into account of the information of control and state *cost* that are critical for evaluating practical control policies.

In this paper, to deal with the above issues, based on probabilistic model checking, we provide an approach for solving optimal finite-horizon control for a CS-PBNp, which considers both random perturbations and cost. Optimal finite-horizon control [1] originates from the field of cancer treatment applications where interventions are typically conducted only for a finite time steps and then are suspended for evaluating the results. The objective of such control is to search for a control policy by manipulating the external control variables that drives the network evolving in a desirable way and minimizes the cost at the same time within the control horizon. In our work, we first describe a detailed procedure of modeling a CS-PBNp using the language provided by a widely used probabilistic model checker PRISM [16], including the steps for describing multiple Boolean functions, context-sensitive switch behavior, and particularly, the effects of random perturbation in a CS-PBNp. We then introduce the reward structure in PRISM for describing quantitative system information. We analyze the reward-based temporal properties and the computation of the properties in probabilistic model checking. Based on the analysis, we provide a method to formulate the optimal control problem as minimum reachability reward properties. Furthermore, we add control and state cost information into the PRISM code of a CS-PBNp such that automated model checking a minimum reachability reward property on the code gives the solution to the optimal control problem.

We conduct experiments on two examples, an apoptosis network [17] and a WNT5A network [15]. The experiment results show the feasibility and effectiveness of our approach. To the best of our knowledge, this is the first time that probabilistic model checking has been used to solve the class of optimal finite-horizon control problem for CS-PBNps. Within the same framework, we also experiment on other variants of finite control problems, in particular, optimal finite-horizon control with multiple hard-constraints [13], which illustrates the flexibility of our approach. These results will be helpful for further utilization of the advances in formal verification techniques to the research of gene regulation.

The rest of the paper is structured as follows: We first briefly introduce the background of context-sensitive PBNs with perturbation and the optimal finite-horizon problem addressed in our work. We then give a detailed account of our approach, including the procedure of describing a CS-PBNp using the modeling language of PRISM and the formulation of the optimal control problem as minimum reachability reward properties, which allows for solving the optimal control problem via automated model checking the properties using PRISM. Finally, we report our experiments on an apoptosis network and a WNT5A network, and discuss the flexibility of the approach.

### Problem outline

In the following, we briefly review context-sensitive probabilistic Boolean networks with perturbation and the optimal finite-horizon control problem investigated in our work; for further details, see [1, 4].

#### Context-sensitive probabilistic boolean networks with perturbation

A Boolean network (BN) on *n* gens is defined by a tuple *B*=(*V*,*F*), where *V*={*x*_1_,…,*x*_*n*_} is a set of nodes – each node *x*_*i*_∈{0,1} (*i*∈[1..*n*]) represents the expression status of the gene *i*, and *F*={*f*_1_,…,*f*_*n*_} is a list of Boolean functions describing the rules of regulatory interactions between genes – each *f*_*i*_:{0,1}^*n*^→{0,1} is the predictor function for the gene *i*. The network state at any time step *t* is given by a *n*-digit binary vector *x*(*t*)=[*x*_1_(*t*)…*x*_*n*_(*t*)], where *x*_*i*_(*t*) is the value of *x*_*i*_ at *t*.

In a probabilistic Boolean network (PBN) [2], each node *i* is associated with multiple candidate Boolean functions denoted by $F_{i} =\{f_{1}^{(i)}, \dots, f_{l(i)}^{(i)}\}$. For each Boolean function $f_{j}^{(i)}$ (*j*∈[1..*l*(*i*)]), the probability of choosing it to update the expression of the gene *i* is given by $c_{j}^{(i)}$ such that $\sum _{j \in [1..l(i)]} c_{j}^{(i)} = 1$. Assume that the governing Boolean functions are chosen independently for each gene. A PBN then represents a set of *N*=*Π*_*i*∈[1..*n*]_ *l*(*i*) number of Boolean networks, and one of then is chosen for deciding the evolution of the PBN with respect to a fixed probability distribution.

To model the inherent stability of biological systems, a *context-sensitive* PBN (CS-PBN) has been developed [4]. The switching between the governing Boolean networks in a CS-PBN is restricted by imposing a binary switch *s* with a small switching probability *q* that forces switching between the underlying Boolean networks. The switching probability *q* is defined as a system parameter: when the value of *s* is 1 with probability *q*, one of the Boolean networks in the CS-PBN is chosen as the evolution rule with respect to the fixed probability distribution; in the case that the value of *s* is 0, the current Boolean network is kept for an interval of time until the next switch.

Moreover, to express the impact of external inputs on the the expression status of gene, random perturbation is incorporated into the structure of a PBN [5]. In this case, each gene is supposed to be perturbed independently with a small probability. Let a binary random variable *p* *e* *r* ^*i*^ (*i*∈[1..*n*]) represent the perturbation of the gene *i* and a parameter *p* be the perturbation probability. When a perturbation occurs, i.e., *p* *e* *r* ^*i*^=1, with the probability *p*, the expression status of the gene *i* flips from 1 to 0 or vice versa. Hence, the PBN corresponds to an ergodic Markov chain, where all the states communicate and there exists a unique steady-state distribution.

In this work, we investigate external control problem of a context-sensitive PBN with perturbation (CS-PBNp), which is believed to be more suitable for modeling small biological subnetworks where the behavior of the genes is moderated by external conditions.

#### Optimal finite-horizon control problem

Optimal finite-horizon control [1] originates from the field of cancer treatment applications where interventions are typically conducted only for a finite time steps and then are suspended for evaluating the results. The goal of such control is to search for a control policy by manipulating the external control variables that drives the network evolving in a desirable way and minimizes the cost at the same time within the control horizon. Assume a CS-PBNp with *n* genes has *m* control inputs, {*u*_1_,…,*u*_*m*_}, and the length of a *finite-horizon* control is *K*. The state of all control inputs at any time step *t* (*t*∈[0..*K*-1]) is represented by a *m*-digit binary vector *u*(*t*)=[*u*_1_(*t*)…*u*_*m*_(*t*)], where *u*_*i*_(*t*) is the value of *u*_*i*_ at *t*. The control-dependent transition probability can be represented by a 2^*n*^×2^*n*^ matrix based on the derivation in [4]. A control policy is represented by *π*={*μ*_0_,…,*μ*_*K*-1_}, where *μ*_*t*_:{0,1}^*n*^→{0,1}^*m*^ denotes a function that maps the state space of the network into the control space. In order to achieve optimal control, given by biologists in practice, a *control cost* function $g_{t}(x(t), u(t)) : \{0,1\}^{n} \times \{0,1\}^{m} \to \mathbb {R}_{\ge 0}$ is used to define the one-step cost of applying the control *u*(*t*) at the state *x*(*t*), and a *state cost* function $g_{K}(x(K)) : \{0,1\}^{n} \to \mathbb {R}_{\ge 0}$ is used to define the terminal cost (or, penalty) for a state *x*(*K*) reached after *K* time steps; in general, based on the desirability in biological applications, a more desirable state is typically assigned a lower terminal cost.

According to the definitions above, given an initial state *x*(0) and a *K*-step control policy *π*={*μ*_0_,…,*μ*_*K*-1_} for a CS-PBNp $\mathcal {P}$, following [1], the expected cost over the control horizon *K*, subject to the control-dependent transition probability in $\mathcal {P}$, is given by

1 $$  \mathcal{J}_{\pi}(x(0)) = E \left[ \sum\limits_{t=0}^{K-1} g_{t}\left(x(t), \mu_{t}(x(t))\right) + g_{K}(x(K)) \right]  $$

The *optimal finite control problem* is to find an optimal control policy $\pi ^{*}=\{\mu ^{*}_{0}, \dots, \mu ^{*}_{K-1}\}$ that minimizes the cost function in Eq. (1).

## Methods

We develope an approach for modeling a CS-PBNp and solving the optimal finite-horizon control problem based on the state-of-the-art probabilistic model checker PRISM [16]. PRISM provides formal techniques for modeling and analyzing systems exhibiting random or probabilistic behavior. Particularly, PRISM provides a simple, state-based language for modeling systems. Based on the code, PRISM can automatically build and analyze the probabilistic models corresponding to the systems, e.g., discrete-time Markov chains (DTMCs), continuous-time Markov chains (CTMCs), Markov decision processes (MDPs), or extensions of these models with reward (alternatively, cost). PRISM supports for analysis of a large family of quantitative properties of the models. The properties are formulated using temporal logics, e.g., Probabilistic Computation Tree Logic (PCTL), Continuous Stochastic Logic (CSL), as well as extensions for quantitative specifications and reward. Thanks to the integration of advanced graph-theoretical and numerical computation algorithms, PRISM can deal with complex and large systems efficiently, and has been widely used for quantitative verification in many different application domains, such as communication protocols, quantum cryptography, and systems biology.

In the rest of this section, we first show how to model a CS-PBNp using the PRISM language. We then introduce the extensions of MDP and PCTL with reward. We analyze computation of reward-based temporal properties. Based on that, we provide a reduction method allowing us to formulate the optimal control problem as a minimum reachability reward property. Furthermore, we add control and state cost information to the PRISM code of a CS-PBNp such that the optimal control problem can automatically solved by model checking the minimum reachability reward property on the code using PRISM.

### Modeling a CS-PBNp in PRISM

Based on the description of a CS-PBN proposed by Kobayashi and Hiraishi [12], we extend with random perturbations, providing a procedure of modeling the complete dynamics of a CS-PBNp using PRISM. We illustrate the procedure using a simple example and then summarize the steps.

Consider a CS-PBNp $\mathcal {P}_{ex}$ composed of two nodes, *x*_1_ and *x*_2_, with one control input *u*, the switching probability *q*=0.3, and the perturbation probability *p*=0.1. The dynamics of $\mathcal {P}_{ex}$ is defined by the following Boolean functions.

2 $$\begin{array}{@{}rcl@{}} x_{1}(t+1) = \left\{ \begin{array}{ll} x_{1}(t) \lor u(t) & c^{(1)}_{1} = 0.3\\ x_{1}(t) \land x_{2}(t) & c^{(1)}_{2} = 0.7 \end{array} \right.  \end{array} $$

3 $$\begin{array}{@{}rcl@{}} x_{2}(t+1)=\left\{ \begin{array}{ll} x_{1}(t) \land \neg u(t) & c^{(2)}_{1} = 0.2\\ x_{2}(t) & c^{(2)}_{2} = 0.8 \end{array} \right.  \end{array} $$

The PRISM code of $\mathcal {P}_{ex}$ is shown as follows.



For the purpose of illustration, we only show the code most related with the node *x*_1_, the control input *u*, context-sensitive switching, and perturbation, defined in different modules. In this code, line 1 shows the system being modeled is an MDP. Lines 2-5 describe the Boolean functions transformed equivalently to numerical expressions on binary values 0 and 1 according to the following rules [11]:

4 $$ \neg x \sim 1-x \;\;\;\;\;\;\; x \lor y \sim x+y-xy \;\;\;\;\;\;\; x \land y \sim xy  $$

For example, lines 2 and 3 are transformed from Eq. (2), and lines 4 and 5 – Eq. (3). Lines 6-9 define the module SWITCH for the the context switch variable s: s is a Boolean variable (line 7) with the probabilities to be *true* (1) and *false* (0) given by 0.3 and 0.7, respectively (line 8). Similarly, lines 10-13 define the module PER1 for the perturbation variable p1, where the probability for *p*1 to be *true* is 0.1, and to be *false* - 0.9. Lines 14-22 describe the module NODE1 for expressing the change of the variable x1 – the status of the node *x*_1_, where the variable d1 records the index of the Boolean function selected for x1 in previous step. In particular, lines 20-21 show that the value of x1 is flipped when the perturbation occurs, i.e., p1=1. Lines 23-27 show the module INPUT for the external input from u, which is non-deterministically assigned 1 and 0, indicating that the control is applied and is absent, respectively. To ensure synchronization over the modules, each command line is attached with the same label [PBN].

Based on the example above, we summarize the steps for deriving the PRISM code of a CS-PBNp as follows.

- **Step 1:** Following the rules in (4), transform each Boolean function $f^{(i)}_{j}$ into an equivalent numerical expression over binary values.
- **Step 2:** Define the PRISM code as an MDP.
- **Step 3:** Define the module SWITCH for the context switch variable *s* as follows, where *q* is the value of the switching probability.

- **Step 4:** Define the module PER *i* for each perturbation variable p *i* as follows, where *p* is the value of the perturbation probability.

- **Step 5:** Define the module NODE *i* for changing the status of each node *x*_*i*_ under context-sensitive switching and perturbation as follows.

- **Step 6:** Define the module INPUT *i* for each control input u *i* as follows.

### Modeling and analysis of optimal control with reward

In the following, we first introduce the reward extensions of MDP and PCTL in PRISM (see [16] for details). We then give a reduction method to formulate the optimal control problems as minimum reachability reward properties. Based on that, we incorporate cost information into the PRISM code derived above such that the optimal control problem can be automatically solved through model checking the properties in the code using PRISM.

#### Extending MDP and PCTL with reward

A reward structure in PRISM describes quantitative reward (or, cost) information of the system that a probabilistic model represents, such as power consumption, execution time, and price of tasks. Consider an MDP $\mathcal {M} = (S, \alpha, \delta)$, where *S* is a finite set of states, *α* is a finite set of actions, and *δ*:*S*×*α*→*D* *i* *s* *t*(*S*) is a partial probabilistic transition function. In PRISM, a reward structure on $\mathcal {M}$ includes two reward functions: the state reward function, $\rho : S \to \mathbb {R}_{\ge 0}$, assigning to each state the reward acquired in the state at each time step, and an action reward function, $\tau : S \times \alpha \to \mathbb {R}_{\ge 0}$, assigning to each pair of (*s*,*a*) the reward acquired when the action *a* is selected in the state *s*.

In PRISM, behavior properties of an MDP can be expressed using PCTL, which provides temporal operators to describe ordering of system events as well as probabilistic quantifiers to specify the likelihood of event occurrence. To express reward-based properties, PCTL is extended with formulas for analyzing quantitative measurement of system models, e.g., instantaneous reward, cumulated reward, and reachability reward. Particularly, for the purpose of our work, we are interested in *reachability reward* properties expressed using the formula R _∼*r*_[F *T*], where ∼∈{<,≤,>,≥}, $r \in \mathbb {R}_{\ge 0}$, F represents the “future” temporal operator, and *T* is a set of target states. R _∼*r*_[F *T*] is true in a state *s* if, from *s*, the expected reward cumulated until the target *T* is reached meets the bound ∼*r*.

For an MDP, due to the existence of the non-deterministic choices of actions, the properties of the *minimum* (or, the *maximum*) expected values of reward are formulated by replacing the R operator with R _min_ (or, R _max_) operator. The meaning of R _min_[F *T*] is: “what is the *minimum expected cumulated reward* of reaching the target *T*?” The value of R _min_[F *T*] is infinite, if, from *s*, the probability of reaching a state in *T* is less than 1 under all possible choices of actions; otherwise, PRISM computes the value based on the following equations:

5 $$ {\begin{aligned} v_{s} = \left\{ \begin{array}{ll} 0 & s \in T \\ \rho(s) + \min\limits_{a\in A(s)}\left(\tau(s,a) + \sum\limits_{s'\in S}\delta(s,a)(s') \cdot v_{s'} \right) & s \notin T \end{array} \right. \end{aligned}}  $$

where *A*(*s*) represents the actions available in *s*.

PRISM integrates a line of numerical algorithms, particularly, dynamic programming techniques based on value iteration, Gauss-Seidel, and policy iteration, for solving linear optimization problems on MDPs, e.g., computing the value of R _min_[F *T*]. Meanwhile, these algorithm are implemented using combinations of symbolic data structures, e.g., multi-terminal binary decision diagrams, and conventional explicit storage engines, e.g., sparse matrices and arrays, achieving compact representations and efficient analysis of complex probabilistic systems.

Moreover, PRISM supports *adversary* generation based on the value iteration algorithm. An adversary represents a possible resolution of all the non-deterministic choices in an MDP, used for choosing actions in each state. An adversary is *deterministic* if only a single action is chosen in each state; an adversary is *memoryless* if the choice of the actions depends only on the current state. For the property R _min_[F *T*], it is known that there always exists an *optimal* adversary, deterministic and memoryless, that gives the minimum expected cumulated reward of reaching a target in *T* [18]; such an optimal adversary can be automatically generated by PRISM.

#### Reduction of optimal control to minimum reachability reward

Reward structures and properties offer effective mechanism for analyzing different quantitative properties. We now show how to formulate the optimal finite-horizon control problem defined in Problem Outline section using the minimum reachability reward property R _min_[F *T*].

Let $\mathcal {P}$ be a CS-PBNp with *n* nodes and *m* control inputs. Consider a finite control of length *K* on $\mathcal {P}$. The dynamics of $\mathcal {P}$ can be defined using an MDP $\mathcal {M}_{\mathcal {P}} = (S, \alpha, \delta)$, where *S*={0,1}^*n*^ is the set states representing the status of the nodes, *α*={0,1}^*m*^ is the set of actions associated with control inputs, and *δ* is the probabilistic transition function defined based on the the control-dependent transition probability for a CS-PBNp [4]. In practice, by making the reward independent of time, the state reward function *ρ* and the action reward function *τ* on $\mathcal {M}_{\mathcal {P}}$ can be generalized from the the state cost function *g*_*K*_ and the control cost function *g*_*t*_ defined for $\mathcal {P}$, respectively.

To solve the optimal control problem for *P* by model checking minimum reachability reward properties, there are issues that need to be addressed. First, the objective of the optimal control problem is to minimize the cost up to a specific time step *K*. The minimum reachability reward property R _min_[F *T*], on the other side, is computed with respect to a set of target states *T*; that is, the information about time step is not taken into account. Second, for the optimal control problem, the cost function defined by Eq. (1) only considers the control cost over the control horizon and the terminal cost in the states reached at the time step *K*; according to Eq. (5), however, R _min_[F *T*] cumulates both the action and the state reward on *every* transition up to the target states.

To address these issues, based on $\mathcal {M}_{\mathcal {P}}$, we construct an MDP $\mathcal {M}'_{\mathcal {P}} = (S', \alpha ', \delta ')$ with new reward functions *ρ* ^′^ and *τ* ^′^, and define a target set *T* ^′^ in $\mathcal {M}'_{\mathcal {P}}$, such that the optimal control problem for $\mathcal {P}$ can be solved by model checking R _min_[F *T* ^′^] over $\mathcal {M}'_{\mathcal {P}}$. The fundamental idea of the construction is to bring in a time step variable *t* to record the progress of the network evolution, which allows us to specify the reward and the new target set *T* ^′^ using the value of *t*. Specifically, the MDP $\mathcal {M}'_{\mathcal {P}} = (S', \alpha ', \delta ')$ is constructed as follows.

- *S* ^′^=*S*×{0,⋯,*K* +1};
- *α* ^′^=*α*;
- for any *s*,*s* ^′^∈*S*, *a*∈*α* ^′^, and *i*≤*K*, *δ* ^′^((*s*,*i*),*a*)((*s* ^′^,*i* +1))=*δ*(*s*,*a*)(*s* ^′^);
- for any *s*,*s* ^′^∈*S*, *a*∈*α* ^′^, *δ* ^′^((*s*,*K* +1),*a*)((*s* ^′^,*K* +1))=*δ*(*s*,*a*)(*s* ^′^).

Furthermore, for the optimal control problem considered here, since Eq. (1) only computes the control cost over the control horizon and the terminal cost in the states reached at the time step *K*, we set zero for other cost in the reward functions on $\mathcal {M}'_{\mathcal {P}}$. Thus, *ρ* ^′^ and *τ* ^′^ are defined as follows.

- for any *s*∈*S* and *i*=*K*, *ρ* ^′^((*s*,*i*))=*ρ*(*s*), and for 0≤*i*≤*K* −1 or *i*=*K* +1, *ρ* ^′^((*s*,*i*))=0;
- for any *s*∈*S*, *a*∈*α* ^′^, and 0≤*i*≤*K* −1, *τ* ^′^((*s*,*i*),*a*)=*τ*(*s*,*a*), and for *K*≤*i*≤*K* +1, *τ* ^′^((*s*,*i*),*a*)=0.

Based on the construction above, solving the optimal finite control problem for $\mathcal {P}$ can be reduced to model checking a minimum reachability reward property on $\mathcal {M}'_{\mathcal {P}}$. Let *s*_*I*_∈*S* be the initial state of $\mathcal {P}$ where the finite control with length *K* starts. We define (*s*_*I*_,0)∈*S* ^′^ be the initial state of $\mathcal {M}'_{\mathcal {P}}$, and specify the target set in *S* ^′^ be *T* ^′^={(*s*,*K* +1)|*s*∈*S*}. According to Eq. (5) and the definitions of the reward functions *ρ* ^′^ and *τ* ^′^, R _min_[F *T* ^′^] computes the minimum expected value of the control cost cumulated over the path to the states in *T* ^′^ and the cost of the states reached at the time step *K*, i.e., {(*s*,*K*)|*s*∈*S*}. Thus, model checking R _min_[F *T* ^′^] gives exactly the optimal value of the cost function defined by Eq. (1) over the control horizon of length *K*.

As introduced before, PRISM can produces an optimal adversary *σ* through model checking R _min_[F *T* ^′^] such that for every state (*s*,*i*) reachable from (*s*_*I*_,0), *σ*((*s*,*i*)) gives a single choice of actions in *α* ^′^. From such an adversary, a control policy *π* for the CS-PBNp $\mathcal {P}$, corresponding the optimal value of the cost function, can be extracted, which is the solution to the optimal control problem.

#### Solving optimal control using PRISM

Based on the analysis above, for a CS-PBNp $\mathcal {P}$, we incorporate time step and cost information into the code derived in Modeling a CS-PBNp in PRISM section. The updated PRISM code corresponds to the MDP $\mathcal {M}'_{\mathcal {P}}$, and therefore can be used for solving the optimal control problem. We illustrate this using the example $\mathcal {P}_{ex}$ again.

Given a finite control over $\mathcal {P}_{ex}$ with the length *K*=4, based on the construction described before, we first define a module STEP for the time step variable *t* as follows.



Next, we describe the sate and control cost information using the reward structures. In PRISM, rewards are described using the following construct:



where *reward* is a real number, *guard* is a predicate characterizing the condition for the states or actions where the *reward* is assigned.

For the example $\mathcal {P}_{ex}$, the cost of the control (with *u*=1) is assumed to be 1, and the terminal cost for the states [0,0], [0,1], [1,0], and [1,1] – 0, 2, 4, and 6, respectively, which implies that the state [0,0] is the most desirable one, and [1,1] – the most undesirable. These information can be added to the previous PRISM code using the reward construct defined as follows.



Line 2 defines the control cost and lines 3-5 define the terminal cost in states. Note that according to the definitions of the reward functions *ρ* ^′^ and *τ* ^′^ discussed previously, we associate the time step variable *t* to the cost definition so that only the control cost in the first 4 steps (*t*∈[0..3]) and the cost of the states reached *after* the 4 steps (*t*=4) are specified; all other ones are set zero by default.

Suppose the finite control over $\mathcal {P}_{ex}$ starts with the initial state *x*[0]=[1,1], i.e., the most undesirable one. For the purpose of illustration, we assume that the switch is turned off at the initial state, there is no perturbation occurring, and the Boolean functions f11 and f21 are chosen for x1 and x2, respectively. Model checking the formula R _min_[F(*t*=5)] with respect to two initial control choices, *u*=1 and *u*=0, returns 4.40 and 4.12, respectively. Thus, the optimal value of the finite control from [1,1] is 4.12. Furthermore, from the optimal adversary generated from PRISM, the following optimal control policy *π*={*μ*_0_,*μ*_1_,*μ*_2_,*μ*_3_} is derived:

- *μ*_0_(*x*[0])=*μ*_1_(*x*[1])=*μ*_2_(*x*[2])=0 for any *x*[0], *x*[1], *x*[3] reached during the first 3 time steps;
- $\mu _{3}(x[3]) = \left \{ \begin {array}{ll} 1 & \text {if}\; x[3] = [1,1] \\ 0 & \text {otherwise} \end {array} \right. $

That is, the control input is applied only in the last time step when the state *x*[3] of the network is equal to [1,1]; otherwise, no control is applied in the policy.

## Results and discussion

We conduct experiments on two examples, an apoptosis network and a WNT5A network, to demonstrate the applicability of our approach on optimal finite-horizon control for CS-PBNp models. We also discuss the flexibility of the approach with experiment on other variants of finite control problems, particularly, optimal finite-horizon control with hard constraints.

### Apoptosis network

Apoptosis, or programmed cell death, is a physiological process allowing an organism to remove damaged or unwanted cells. The interactions within this process is fundamental for embryonic development and adult organisms. Malfunctioning apoptosis network may lead to various diseases, such as cancer, or immunodeficiency and infertility. For the demonstration purpose of this paper, we construct a CS-PBNp for an apoptosis network adapted from [17], and apply our approach to solving the optimal control problems for the network.

The apoptosis network used in our experiments consists of one control input: the concentration level of TNF denoted by *u*, and six nodes: the concentration level of IAP denoted by *x*_1_, the concentration level of C3a by *x*_2_, the concentration level of C8a by *x*_3_, the concentration level of NF *κ* *B*_*nuc*_ by *x*_4_, the concentration level of NF *κ*B by *x*_5_, and the concentration level of CARP by *x*_6_. According to [17], TNF is used as an control input to the system – its stimulation triggers two opposite effects: activation and inhibition of caspases. The dynamics induced by the underlying pro- and anti-apoptotic genes will lead to different states of cell death or cell survival. A high concentration of IAP and a low level of active caspases, e.g., C3a, typically characterizes a living cell, whereas a high level of active caspases together with a low concentration of IAP typically leads to cell death. From this observation, all the states where *x*_1_=1 and *x*_2_=0 are treated as the most desirable states for regulation purpose, which are assigned a state penalty of 0; the states where *x*_1_=0 and *x*_2_=1, the most undesirable ones, are assigned a penalty of 10; and other states – a penalty of 5. The cost of control is assumed to be 1, with *u*=1 signifying TNF is activated and *u*=0 – TNF is absent. To attain a CS-PBNp, we adopt synchronous and asynchronous update [11] to extend the dynamics of the network, building eight Boolean networks used as the constituent Boolean networks. The probabilities for context-switch and perturbation are set *q* = 0.3 and *p* = 0.1, respectively.

Following our approach developed in the paper, the CS-PBNp is modeled using PRISM code. We consider finite-horizon control of four different lengths: 4, 6, 8, and 10, and experiment with six different initial states where the evolution of the network starts: *s*_0_=[100111], *s*_1_=[101111], *s*_2_=[000111], *s*_3_=[111111], *s*_4_=[010111], and *s*_5_=[011111]. The last three bits are set the same for meaningful comparison of the experiment results. Note that *s*_0_ and *s*_1_ are the most desirable states, and that *s*_4_ and *s*_5_ – the most undesirable ones. For modeling stability of biological systems, we assume no context switch and no perturbation at the initial states, and assign them a default Boolean network.

**Fig. 1:**
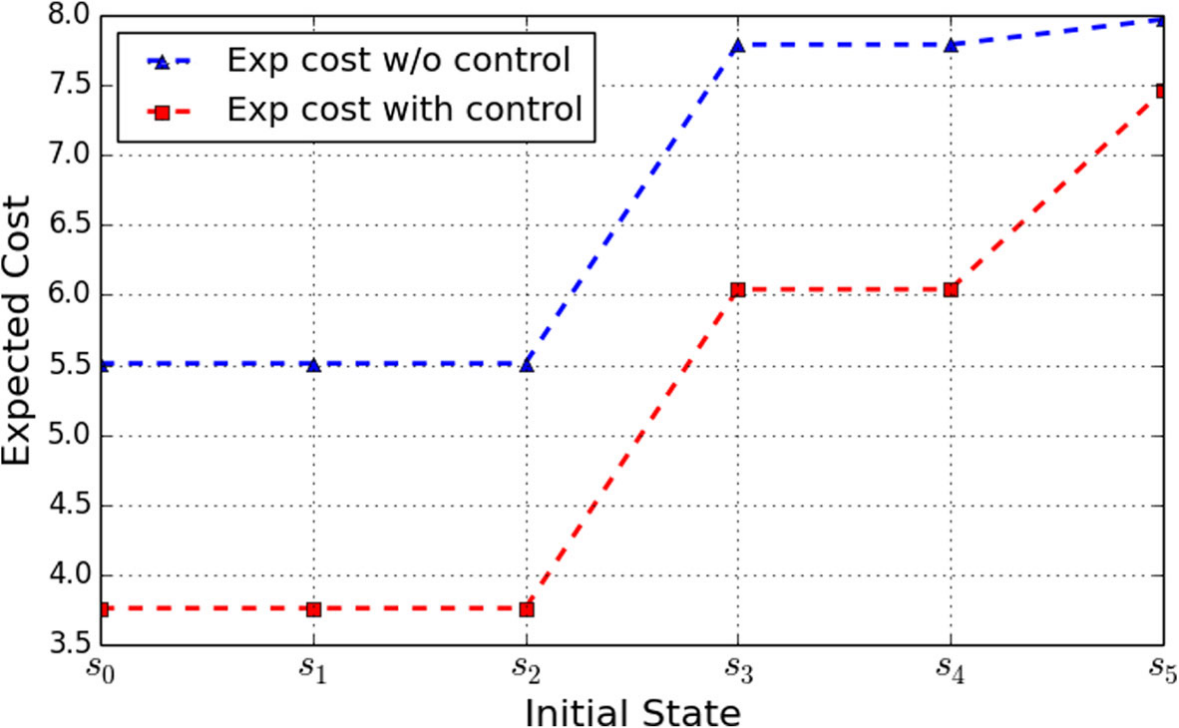
Apoptosis Network: Expected cost for a finite-horizon problem of length 4 originating from the different initial states

**Fig. 2:**
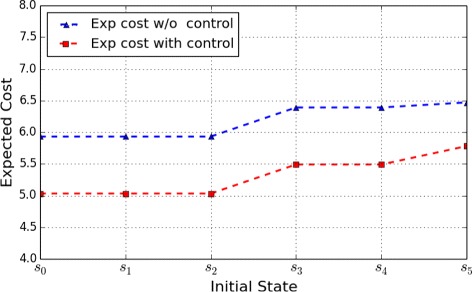
Apoptosis Network: Expected cost for a finite-horizon problem of length 10 originating from the different initial states

Figures 1 and 2 show the optimal expected cost with control from different initial states, as well as the expected cost without control, under the control horizon of lengths 4 and 10, respectively. The objective of this control is to shift the network toward desirable states – cell survival states – that are assigned a lower penalty. According to the figures, we can see that the expected cost with control is lower than that without control, which is consistent with the control objective.

**Fig. 3:**
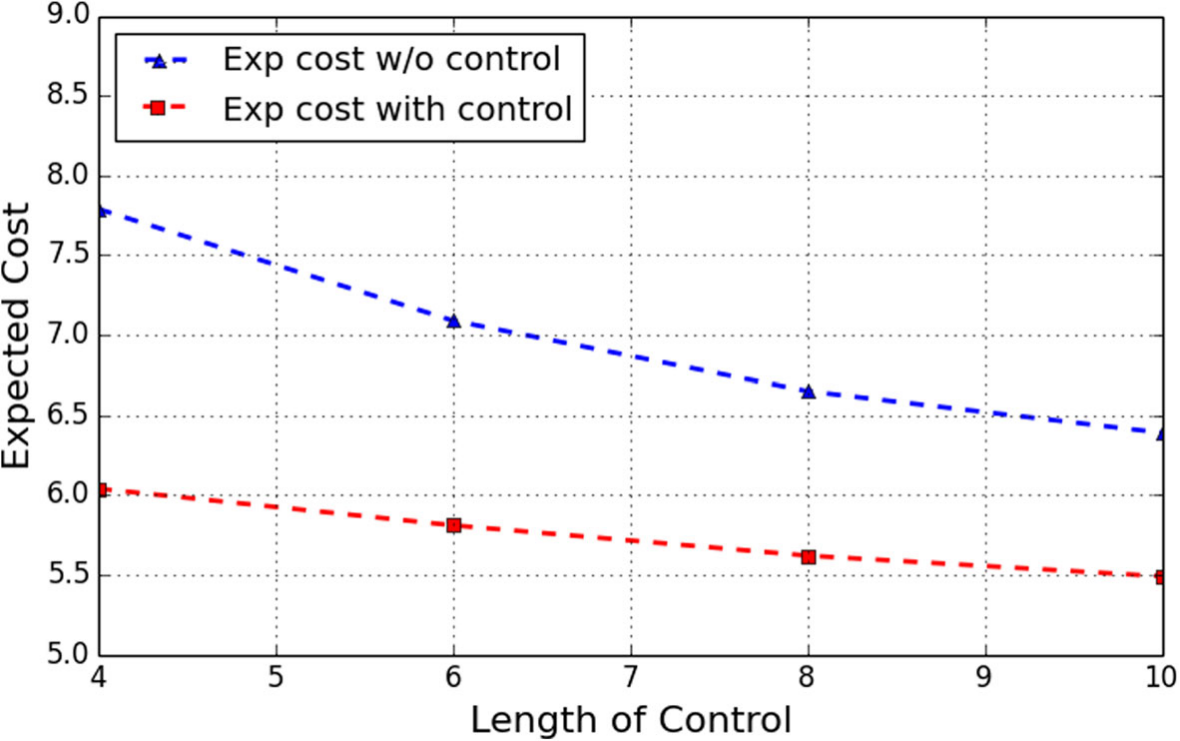
Apoptosis Network: Expected cost of finite-horizon control of different lengths originating from the initial state *s*_4_

**Fig. 4:**
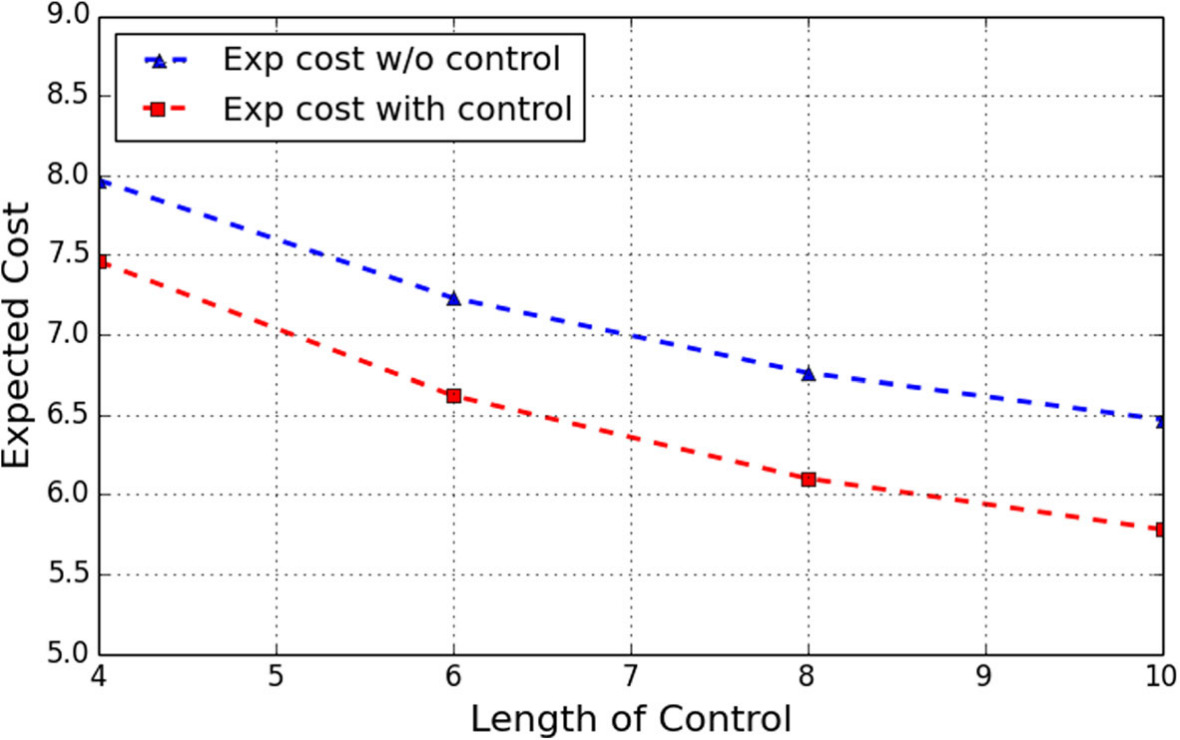
Apoptosis Network: Expected cost of finite-horizon control of different lengths originating from the initial state *s*_5_

Moreover, Figs. 3 and 4, respectively, show the expect cost from the two most undesirable initial states, *s*_4_ and *s*_5_, under different lengths of control. From the figures, it can be observed that with the increase of the length of control horizon, the optimal cost of the control tends to gradually decrease to a steady value.

**Fig. 5:**
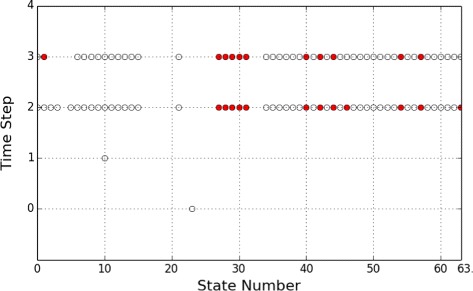
Apoptosis Network: Optimal control policy for the horizon length 4 originating from the initial state *s*_4_

Finally, Fig. 5 presents the optimal control policy for the finite-horizon control of length 4 starting from the initial state *s*_4_, where state numbers are represented using the decimal equivalent of the binary encoding of each state, a red circle indicates that the control input is applied, and a white circle – no control. The policy is extracted from the optimal adversary generated from PRISM, and the control decisions shown here are only for the states reachable from *s*_4_ in the 4 time steps. It can be seen from the figure that the optimal control policy is not to apply control input for the first two time steps (*t*=0 and *t*=1) – note that because one default Boolean network is assigned to the initial state *s*_4_, only one state is reached at the second time step; control inputs are applied in the last two time steps (*t*=3 and *t*=4).

### WNT5A network

A WNT5A network is a gene regulatory network developed from the study of metastatic melanoma [19], where using an intervention to downregulate the WNT5A gene’s action could decrease the possibility of a melanoma metastasizing. Following [15, 20], we consider a WNT5A network consisting of seven genes: WNT5A denoted by *x*_1_, pirin by *x*_2_, S100P by *x*_3_, RET1 by *x*_4_, MART1 by *x*_5_, HADHB by *x*_6_, and STC2 by *x*_7_. Since the control objective is to keep WNT5A downregulated, the most desirable states are the ones where *x*_1_=0, which are assigned a penalty of 0, and the most undesirable states, with *x*_1_=1, are assigned a penalty of 5. Moreover, according to [15], the control strategies of a WNT5A network are designed by choosing pirin as the control gene, with the control input *u*=1 indicating that the state of pirin is reversed and *u*=0 indicating no external intervention. A control cost of 1 is incurred when the control action forces the state of pirin be reversed. We obtain a CS-PBNp of a WNT5A network through synchronous and asynchronous update, constructing four Boolean networks used as the constituent Boolean networks. The probabilities for context-switch and perturbation are defined by *q*=0.1 and *p*=0.2, respectively.

For the WNT5A network described above, we consider finite-horizon control of five different lengths: 4, 6, 8, and 10. We also experiment with six different initial states where the evolution of the network starts, i.e., *s*_0_=[0000000], *s*_1_=[0111110], *s*_2_=[0110110], *s*_3_=[0011110], *s*_4_=[1000001], *s*_5_=[1000100], *s*_6_=[1010001], and *s*_7_=[1111010]. Note that the first three states are desirable states, whereas the last three ones are undesirable ones. At the initial states, we assume that there is no context switch and no perturbation, and assign a default Boolean network to them.

**Fig. 6:**
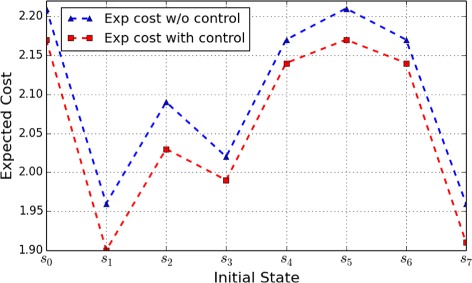
WNT5A Network: Expected cost for a finite-horizon problem of length 8 originating from the different initial states

**Fig. 7:**
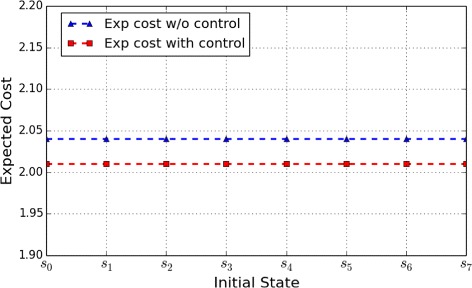
WNT5A Network: Expected cost for a finite-horizon problem of length 16 originating from the different initial states

Figures 6 and 7, respectively, show the expected cost for a finite horizon problem of length 8 and length 16, starting from the different initial states. The goal of the control is to reduce the activity of WNT5A gene in affecting biological regulation. We can see from the two figures that the expected cost with control is lower than that without control, which agrees with the objective.

Furthermore, comparing Figs. 6 and 7 indicates that with the increase of the length of control horizon, the difference between the expected cost obtained from different initial states narrows – the initial states yield almost the same expected cost. The same observation is also reported in [15]; this may be caused by limited level of the underlying networks and the existence of random perturbation in the corresponding ergodic Markov chain.

**Fig. 8:**
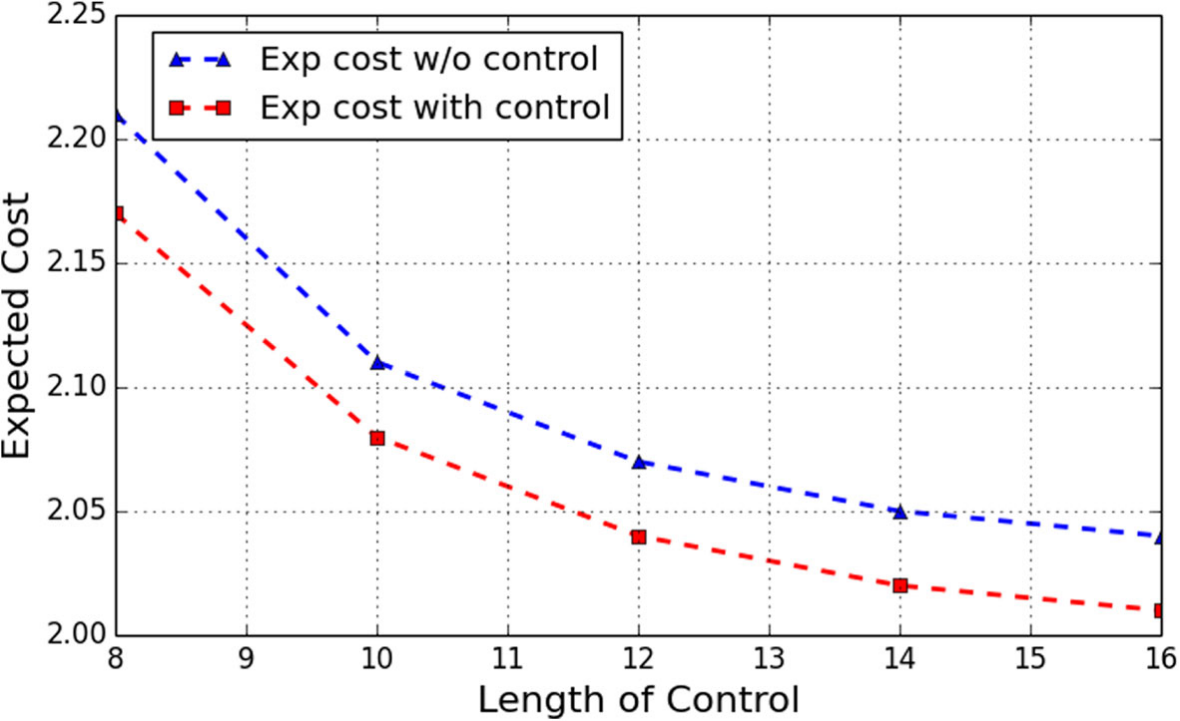
WNT5A Network: Expected cost of finite-horizon control of different lengths originating from the initial state *s*_5_

**Fig. 9:**
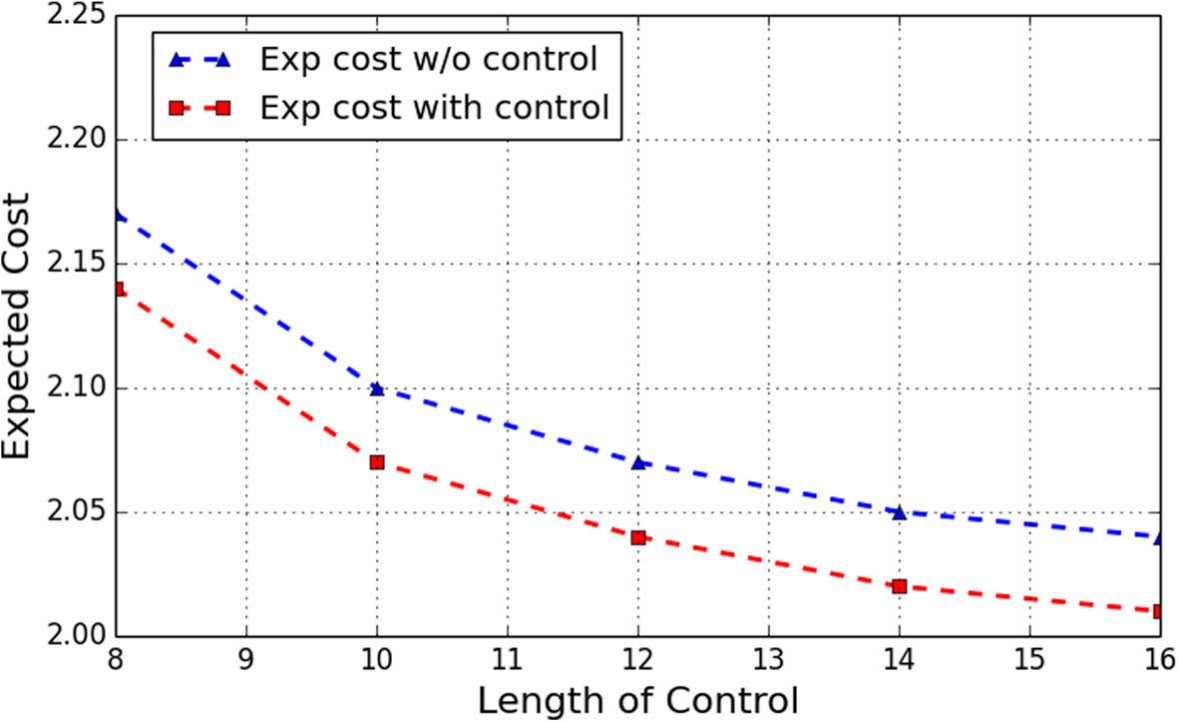
WNT5A Network: Expected cost of finite-horizon control of different lengths originating from the initial state *s*_6_

Figs. 8 and 9, respectively, show the expect cost from the two most undesirable initial states, *s*_5_ and *s*_6_, under different lengths of control. Similar to that observed in the apoptosis network, with the increase of the length of control horizon, the expected cost of the control also tends to gradually decrease to a steady value.

**Fig. 10:**
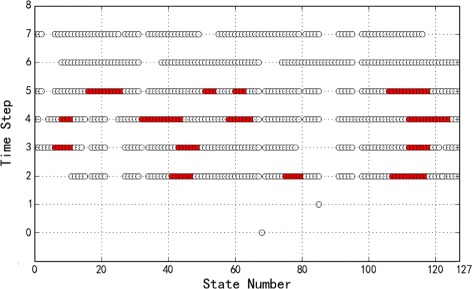
WNT5A Network: Optimal control policy for the horizon length 8 originating from the initial state *s*_5_

Figure 10 shows the optimal control policy for the finite-horizon control of length 8 starting from the initial state *s*_5_, where state numbers are represented using the decimal equivalent of the binary encoding of each state, a red circle implies that the control input is applied, and a white circle – no control. From the figure, it can be seen that the optimal control policy is to apply control from the third step (*t*=2) to the sixth step (*t*=5) depending on the states.

### Discussion

In this paper, we have focused on the optimal finite-horizon control problem defined in Problem Outline section. On the other side, as observed in our work, a model checking-based approach enables a natural depiction of the dynamics of gene regulatory networks, and provides a canonical form to formulate control problems using temporal logic formulas that can be automated solved by leveraging the analysis power of underlying model checking engines. Given these features, our approach, in fact, has the flexibility to solve other variants of optimal control problems within the same framework.

For example, for the optimal control problem defined in Problem Outline section, suppose an upper bound is imposed for the number of controls that can applied to a network during the entire control horizon, which is referred as *optimal finite-horizon control with multiple hard-constraints* [13]; such constraints are important for medical applications where the maximum number of treatments, e.g., radiation and chemo-therapy, is restricted. In this case, suppose the control horizon is *K*, the objective of optimal control is to minimize $\mathcal {J}_{\pi }(x(0))$ subject to the constraints 0≤*#* *u*_*i*_≤*H*_1_,..., and 0≤*#* *u*_*m*_≤*H*_*m*_, where $\mathcal {J}_{\pi }(x(0))$ is the cost function defined in Eq. (1), *#* *u*_*i*_ is the number of times that the control *u*_*i*_ is applied, and *H*_*i*_ (*H*_*i*_∈[0..*K*]) is the upper bound of *#* *u*_*i*_.

Within the framework of our approach, this problem can be solved as follows. To record the number of controls applied, we add to the previous PRISM code a variable *c* *t*_*i*_ for each control input *u*_*i*_: *c* *t*_*i*_ is initialized 0, and increases by 1 at each time step if and only if *u*_*i*_=1. Then, the optimal control problem with the constraints can be reduced to model checking the minimum reachability reward property given by the formula $\text {\texttt {R}}_{\text {\texttt {min}}}\Big [{\text {\texttt {F}}} {\Big ((t \leq K\mathtt {+}1) \wedge (ct_{1} \leq H_{1}) \wedge \dots \wedge (ct_{m} \leq H_{m}) \Big) }\Big ]$.

Consider the apoptosis network in previous experiments and the finite control with length 6 starting from the initial state *s*_4_. Suppose we add the constraint that the maximum number of controls applied within the control horizon is 3. Based on the steps above, we obtain that the optimal cost under this constraint is 6.03, which is higher than the optimal value without the constraint – 5.81 (Fig. 3). Similarly, for the WNT5A network, consider the finite control starting from the initial state *s*_5_ with length 8. Suppose we add an upper bound 3 over the maximum number of controls applied within the control horizon. We obtain that the optimal cost in this case is 2.18 as compared with 2.17 (Fig. 8) for no such constraint.

The discussion above shows the flexibility of our approach. Following this, we would like to evaluate our approach on more biological networks in future, exploring a wider class of optimal control problems that our approach can solve. Meanwhile, to further exploit the modeling and analysis power of the method based on model checking, it seems interesting to investigate combination of probabilistic model checking and optimization approaches, e.g., genetic algorithms, for larger-scale networks; the idea is to use probabilistic model checker as a solver for cost functions, while using the heuristic mechanisms in other approaches to guide the search for control strategies.

## Conclusions

In this paper, we studied optimal finite-horizon control for probabilistic gene regulatory networks based on PBNs. Specifically, we proposed an approach for solving the problem for CS-PBNps using probabilistic model checking. We presented a procedure of modeling a CS-PBNp using PRISM code. Based on analysis of reward structures and properties, we reduced the optimal control problem to automated model checking minimum reachability reward properties. Preliminary experiment results show the feasibility and effectiveness of our approach. The approach based on probabilistic model checking for optimal control avoids explicit computation of large-size state transition relations associated with PBNs. It also support convenient modeling of gene regulatory networks and specification of optimal control problems using temporal properties that can be automated solved by the state-of-the-art model checkers. We hope that our work can be helpful for further application of advanced formal verification techniques in system biology.

## Abbreviations

BN: Boolean network
CS-PBN: Context-sensitive PBN
CS-PBNp: Context-sensitive PBN with perturbation
CSL: Continuous stochastic logic
CTMC: Continuous-time Markov chain
DTMC: Discrete-time Markov chain
MDP: Markov decision process
PBN: Probabilistic Boolean network
PCTL: Probabilistic computation tree logic
